# microRNA‐148a‐3p in extracellular vesicles derived from bone marrow mesenchymal stem cells suppresses SMURF1 to prevent osteonecrosis of femoral head

**DOI:** 10.1111/jcmm.15766

**Published:** 2020-09-01

**Authors:** Shengxiang Huang, Yaochun Li, Panfeng Wu, Yongbing Xiao, Ningbo Duan, Jing Quan, Wei Du

**Affiliations:** ^1^ Department of Orthopedics Hunan Children's Hospital Changsha China; ^2^ Department of Rehabilitation Xiangya Hospital of Central South University Changsha China; ^3^ Department of Orthopedics Xiangya Hospital of Central South University Changsha China

**Keywords:** BCL2, bone marrow mesenchymal stem cells, extracellular vesicles, microRNA‐148a‐3p, osteonecrosis of the Femeral Head, SMAD7, SMURF1

## Abstract

Extracellular vesicle (EV)‐associated microRNAs (miRNAs) have been found as the important biomarkers participating in the development of osteonecrosis of the femoral head (ONFH). Consequently, this study sought to examine the underlying mechanism of bone marrow mesenchymal stem cell (BMSC)‐derived EVs containing miR‐148a‐3p in ONFH. The ONFH rat models were established. Reverse transcription quantitative polymerase chain reaction (RT‐qPCR) and Western blot analysis were applied to detect miR‐148a‐3p, Smad ubiquitination regulatory factor 1 (SMURF1), SMAD7 and B‐cell CLL/lymphoma 2 (BCL2) expression, followed by determination of relationship between miR‐148a‐3p and SMURF1. BMSCs were isolated from normal rats and ONFH rats, and EVs were extracted from BMSCs of normal rats. BMSCs from ONFH rats were treated with mimic, inhibitor, small interfering RNA or EVs from miR‐148a‐3p mimic‐treated BMSCs from normal rats (BMSC‐EV‐miR‐148a‐3p mimic). Cell Counting Kit‐8 and alizarin red staining were utilized to detect cell viability and osteogenic differentiation of BMSCs. ONFH rats were injected with BMSC‐EV‐miR‐148a‐3p mimic to explore the function of BMSC‐EV‐delivered miR‐148a‐3p in vivo. miR‐148a‐3p was down‐regulated in BMSCs and EVs from ONFH rats following decreased BMSCs viability and osteogenic differentiation. SMURF1 was a target gene of miR‐148a‐3p, and resulted in ubiquitination and degradation of SMAD7 to decreased BCL2 expression. The proliferation and differentiation of BMSCs were promoted by BMSC‐EV‐miR‐148a‐3p mimic or SMURF1 silencing. Additionally, BMSC‐EV‐miR‐148a‐3p mimic increased cell proliferation and osteogenic response, diminished SMURF1 expression, and elevated SMAD7 and BCL2 expression in ONFH rats. Collectively, miR‐148a‐3p overexpressed in BMSC‐EVs promoted SMAD7 and BCL2 expression by inhibiting SMURF1, thus alleviating ONFH.

## 1 INTRODUCTION

Osteonecrosis of the femoral head (ONFH), as one of debilitating diseases, results in the collapse of femoral head and further evolves into degenerative arthritis.^1^ The patients suffering from ONFH have poor prognosis.^2^ At present, the treatment and clinical results are not perfect in ONFH.^3^ Notably, there is the decrease of the proliferation and osteogenesis as well as the increase of apoptosis and adipogenesis in bone marrow mesenchymal stem cells (BMSCs) from patients with ONFH.^4^ Therefore, this study attempts to research a new therapeutic method for ONFH based on BMSCs.

Mesenchymal stem cells (MSCs) have the capacity to repair the injured tissues by the activity of paracrine, among which extracellular vesicles (EVs) are considered as the process of reacted mechanism in MSCs.^5^ In addition, EVs are made up of exosomes (30‐150 nm) and microvesicles (100‐1500 nm), functioning in the regulation of the communication among cells.^6^ It was reported that BMSC‐derived EVs prevented femoral head necrosis.^7^ There are many proteins, messenger RNAs (mRNAs) and microRNAs (miRs) in BMSC‐EVs, functioning in the diverse biological progress.^8^ Moreover, EVs derived from BMSCs were detected to contain miR‐148a.^9^ miR‐148a‐3p is poorly expressed in ONFH, involving in the osteogenic differentiation of BMSCs.^10^ Then, the binding sites between miR‐148a‐3p and SMURF1 3′untranslated region (3′UTR) were predicted by StarBase in our study. SMURF1 has the homology with E6AP C‐terminus‐type E3 ubiquitin ligase, thereby being involved in bone morphogenetic protein and remodelling.^11^ Furthermore, down‐regulated Smad ubiquitination regulatory factor 1 (SMURF1) is found to promote the expression of SMAD7 in mesangial cells.^12^, ^13^ SMAD7 is promoted by the interaction between microtubule actin crosslinking factor 1 and SMAD7 to accelerate the osteogenesis.^14^ Furthermore, up‐regulated SMAD7 is closely related to the proliferation of BMSCs.^15^ SMAD7 elevates the expression of B‐cell CLL/lymphoma 2 (BCL2).^16^ EVs that are derived from human platelet‐rich plasma promote the expression of BCL2 to prevent the cell apoptosis in ONFH.^17^ At present, few studies have explored the combined regulatory role of BMSC‐EV‐miR‐148a‐3p/SMURF1/SMAD7/BCL2 axis in ONFH. This study attempted to investigate the relationship among miR‐148a‐3p, SMURF1, SMAD7 and BCL2 in ONFH and the related mechanisms.

## 2 MATERIALS AND METHODS

### 2.1 Ethics statement

Study protocols were approved by Ethic Committee of Xiangya Hospital of Central South University. The animal experiments were performed in accordance with the recommendations in the *Guide for the Care and Use of Laboratory Animals*. The protocol of animal experiments was approved by the Ethic Committee of Xiangya Hospital of Central South University.

### 2.2 Cell culture and transfection

The tibia and femur of 2 Sprague‐Dawlay (SD) rats (male) were aseptically obtained, and then the connective and muscle tissues were removed to be conducive to cutting both ends of the bone with scissors. The needle (No. 5) was utilized to extract the bone marrow cavity, which was repeatedly washed with serum‐free dulbecco's modified eagle medium (DMEM) and made into a single‐cell suspension. The suspension was centrifuged at 900 *g* for 5 minutes. After that, the collected mixture suspension was resuspended at a ratio of 10^5^/cm^2^ in a culture bottle containing DMEM with 10% foetal bovine serum (FBS) (Gibco), 100 μg/mL streptomycin and 100 μg/mL penicillin (Gibco), and then cultured in a 5% CO_2_ incubator at 37°C to passage for later use.

miR‐148a‐3p mimic and its negative control (NC) plasmids were purchased from GenePharma Technology Co., Ltd. Cells were transfected with 100 nmol/L miR‐148a‐3p mimic or its NC plasmids using Lipofectamine 2000 reagent according to the protocols (Invitrogen). After the transfected cells were incubated with 5% CO_2_ at 37°C for 6‐8 hours, they were placed in a complete medium for 24‐48 hours for the subsequent experiments.

### 2.3 Cell viability assay

MSCs were inoculated into a 96‐well plate at a density of 5 × 10^3^ cells/well. The initial isolation medium was aspirated and renewed with complete medium after cell adhesion. The cell viability was determined by Cell Counting Kit‐8 (CCK‐8) assay at designated time points in accordance with the instructions.

### 2.4 Oil red O staining

MSCs were washed twice with phosphate buffer saline (PBS) and fixed with 4% formaldehyde for 30 minutes at room temperature. Subsequently, after staining with filtered oil red O solution at room temperature for 1 hour, the cells were observed and photographed under a light microscope. To extract oil red O, each well was added with 1 mL of isopropanol and oscillated at room temperature for 15 minutes. The absorbance of 3 samples at 490 nm was recorded after proper dilution.

### 2.5 Alkaline phosphatase staining

MSCs were also washed twice with PBS and fixed with 4% formaldehyde for 30 minutes at room temperature. Next, after washing with PBS three times, the cells were cultured with 5 mL of staining buffer (100 mmol/L Tris HCl, 150 mol/L NaCl and 1 mmol/L MgCl_2_) for alkaline phosphatase staining. The staining buffer contained colouring substrate solution which consisted of 33 μL nitroblue tetrazolium (NBT) (50 mg/mL) and 16.5 μL of 5‐bromo‐4‐chloro‐3‐indolyl phosphate (BCIP) (50 mg/mL). The cells were stained with BCIP/NBT matrix for 30 minutes. After the matrix solution was removed, the cells were washed with deionized water, and then observed under a light microscope and photographed.

### 2.6 Alizarin red staining

MSCs were also washed twice with PBS and fixed with 4% formaldehyde for 30 minutes at room temperature. Then, the cells were stained with a 40 mmol/L ARS solution (pH = 4.2) for 20 minutes after washing twice with PBS. The cells were washed with PBS five times to reduce non‐special staining. Osteogenic differentiation was quantified by measuring the area stained with Alizarin red using Meta Morph imaging software (Universal Imaging, Downingtown, Pennsylvania, USA). Alizarin red staining was quantified using Image pro‐plus to calculate the osteogenic integral optical density in the experiments in vivo.

### 2.7 Isolation and identification of EVs

When reaching 80% confluence, the surface‐adherent cells were washed with PBS and incubated in MesenGro‐MSC medium (StemRD) without FBS for 48 hours. The conditioned medium was obtained, and then centrifuged at 300 *g* for 10 minutes and at 2000 *g* for 10 minutes to remove the dead cells and cell debris. After that, the supernatant was filtered with a 0.22 μmol/L filter (micropore) and centrifuged in Amicon ultra‐15 mL centrifugal filter device (micropore) at 4000 *g* to roughly 200 μL by ultrafiltration. The ultrafiltrate was washed twice with PBS and ultrafiltered again at 4000 *g*.

For EV purification, the liquid was covered with 30% sucrose‐dehydrated water buffer, placed in a sterile ultra‐transparent test tube (Beckman Coulter, Brea, CA, USA), and ultracentrifuged for 1 hour at 100 000 *g*. Subsequently, granular EVs were resuspended with PBS and centrifuged at 4000 *g* to about 200 μL. All procedures were performed at 4°C. EVs were stored at −80°C or used for subsequent experiments.

The collected EVs were identified by dynamic light scattering (DLS), transmission electron microscopy (TEM) and Western blot analysis. The size distribution of EVs was determined by DLS analysis using Nanosizer™ instrument (Malvern Instruments, Malvern, UK). The samples were diluted with filtered Dulbecco's PBS (1000×). In addition, the data processing and analysis were performed on Zetasizer software (Malvern). The morphology of EVs was observed by TEM. After that, EVs were fixed with 2% paraformaldehyde solution, cut into ultrathin sections and labelled with anti‐CD63 antibodies (ab108950, Abcam, Cambridge, UK). All sections were examined, recorded and photographed under a TEM (H‐7650; Hitachi, Tokyo, Japan). The surface markers of CD63‐EVs were analysed by Western blot analysis with ultrafiltrate as the NC of Western blot analysis.

### 2.8 EV internalization

EVs produced by MSCs were labelled with Dil dye (Molecular Probes, Eugene, OR, USA) following the instructions. Briefly, the cells were trypsinized and resuspended in 1 mL of serum‐free α‐minimum Eagle's medium. A total of 5 mL cell labelling solution was added to the cell culture medium and incubated with cells at 37°C for 15 minutes. The cell‐labelled suspension was centrifuged at 300 *g* for 15 minutes, and the supernatant was discarded. After that, the cells were washed with PBS and cultured in MesenGro‐MSC medium (StemRD) for 24 hours. The extracted EVs were cultured with BMSCs at 37°C for 2 hours. Then, BMSCs were fixed with 4% paraformaldehyde for 15 minutes and stained with 4′,6‐diamidino‐2‐phenylindole (DAPI) staining kit for 5 minutes. The internalization of EVs was observed by fluorescence microscope (Leica DMI6000B, Solms, Germany).

### 2.9 Western blot analysis

EVs in cell supernatants were lysed with Radio Immunoprecipitation Assay (RIPA) lysis buffer (Promega, Madison, WI, USA) containing protease inhibitors. The extract solution of whole cell protein was homogenized in RIPA buffer lysis buffer (Promega), centrifuged at 10,000 × g for 20 minutes, and determined by bicinchoninic acid using a commercial kit (Thermo Fisher Scientific, Waltham, MA, USA). The proteins were transferred to the nitrocellulose filters (Millipore, Billerica, MA, USA) and incubated with specific primary antibodies such as rabbit anti‐CD63 (ab134045, 1:1000, Abcam), SMAD7 (ab216428, 1:500, Abcam) and BCL2 (ab32124, 1:1000, Abcam). After washing with Tris‐buffered saline Tween three times, nitrocellulose filters were incubated with horseradish peroxidase‐conjugated goat anti‐rabbit secondary antibody immunoglobulin G (IgG) (1:2000, ab6721, Abcam) at room temperature for 2 hours. Protein bands were detected by ChemiDoc XRS + chemiluminescence imaging system (Bio‐Rad, Hercules, CA, USA). The relative protein content was expressed as the grey value of the corresponding protein band/the glyceraldehyde‐3‐phosphate dehydrogenase (GAPDH) protein band.

### 2.10 Reverse transcription quantitative polymerase chain reaction (RT‐qPCR)

RNA was extracted and quantified by Trizol or Trizol LS (Thermo Fisher Scientific) and RT‐qPCR. The primer sequences used in RT‐qPCR are shown in Table 1. U6 small nuclear RNA was used as an internal control to detect intracellular miRNA and 20 fmoL of synthetic cel‐miR‐39‐3p was added to EVs from an equal number of cells during RNA extraction to detect miRNA in EVs. The PCR instrument was purchased from Bio‐Rad. As for the quantification of intracellular mRNA, GAPDH was used as an internal control. The exponential relationship between the expression of the target gene in the experimental and control groups was calculated by 2^−ΔΔCt^. The formula was ΔΔCT = ΔCt experimental group ‐ ΔCt control group (ΔCt = Ct target gene ‐ Ct reference gene). At this time, the amplification was in a logarithmic phase.

**Table 1:** Primer sequences for RT‐qPCR

Primer sequences	Forward (5′‐3′)	Reverse (5′‐3′)
cel‐miR‐39	GGTCACCGGGTGTAAATCAGCTTG	/
miR‐148a‐3p	CTCAACTGGTGTCGTGGAGTCGGCAATTCAGTTGAGCAAAGTTC	GCCGAGTCAGTGCACTACA
U6	AACGCTTCACGAATTTGCGT	CTCGCTTCGGCAGCACA
SMURF1	CTACCAGCGTTTGGATCTAT	TTCATGATGTGGTGAAGCCG
GAPDH	ATCCCATCACCATCTTCC	GAGTCCTTCCACGATACCA

Abbreviations: GAPDH, Glyceraldehyde‐3‐phosphate dehydrogenase; miR, microRNA; RT‐qPCR, reverse transcription quantitative polymerase chain reaction; SMURF1, Smad ubiquitination regulatory factor 1.

### 2.11 Luciferase activity assay

HEK293T cells (CRL‐1415, Xinyu Co., Ltd., Shanghai, China) were transfected with psiCHECK2 reporter plasmid constructs (Promega) containing wild‐type (WT) or mutant‐type (MUT) SMURF1 3′UTR, which then was co‐transfected with miR‐148a‐3p mimic or NC plasmids. The luciferase activity was detected by dual‐luciferase kit (Promega) in line with the instructions of manufacturer after 48 hours with renilla luciferase activity as an internal reference. The relative luciferase activity = Firefly luciferase activity/Renilla luciferase activity.

### 2.12 Ubiquitination analysis

BMSCs were treated with 10 μmol/L of MG132 (MedChemExpress, NJ, USA) for 4 hours, lysed with the conventional lysis buffer (100 μL), and denatured at 95°C for 5 minutes in the presence of 1% sodium dodecyl sulphate. After that, the cell lysate was incubated with anti‐Krüppel‐like factor 2 antibody and protein G agarose (Sigma, St. Louis, MO, USA) (www.sigmaaldrich.com) at 4°C overnight. The anti‐ubiquitin antibody was analysed by Western blot analysis to detect endogenous ubiquitination of SMAD7.

### 2.13 Animal models and grouping

A total of 36 female SD rats (200‐210 g) were enrolled in this study and equally divided into 6 types of rats such as the normal rats, induced ONFH rats and ONFH rats respectively injected with PBS, EVs, EVs and mimic‐NC plasmids, and EVs and miR‐148a‐3p mimic plasmids. To induce ONFH rats, methylprednisolone (MPS; Pfize, NY, USA) (20 mg/kg/d) was intramuscularly injected into rats on the first 3 days of every week for 3 weeks. After injecting with MPS, the model rats were injected with 1 × 10^11^ particles of EVs (dissolved in 200 μL of PBS) or an equal volume of PBS via the vein of tail. After 6 weeks, the rats were anaesthetized with 1 mg/kg of sodium pentobarbital by peritoneal injection.^17^ The femoral head was observed by micro‐Computed Tomography (CT) and analysed by immunohistochemistry and histology. In all experimental rats, none of rats died before these assessments and no antibiotics were used throughout the study.

### 2.14 Micro‐CT analysis

The femoral heads were dissected from the rats, fixed in formalin overnight and analysed by SkyScan 1178 (Bruker MicroCT, Kontich, Belgium). The resolution of the scanner was set to 9 μmol/L per pixel. The trabecular bones were isolated from bone marrow and analysed to determine the trabecular thickness (Tb.Th), trabecular separation (Tb.Sp), bone volume/tissue volume (BV/TV) and trabecular number (Tb.N). Three planes (coronal, sagittal and transect planes) in each representative sample were analysed by a data viewer (vivaCT80; SCANCO Medical, Switzerland).

### 2.15 Histological and immunohistochemical analysis

The femoral heads were fixed with 10% formalin buffer for 24 hours and decalcified with 10% ethylenediaminetetraacetic acid for 28 days. Then the heads were embedded with paraffin, cut into 5 μmol/L sections, dewaxed with xylene, rehydrated with the graded ethanol and rinsed with distilled water. After that, the heads were stained with haematoxylin‐eosin staining and observed by histology analysis.

The cell proliferation was measured by Ki67 antibody (1:500, ab15580, Abcam). The cell nucleus was stained with DAPI and the images were obtained by LSM‐880 confocal microscope (Carl Zeiss, Oberkochen, Germany). The Ki67 positive cells (proliferative cells) in each area were evaluated in 5 areas per section and 5 areas per femoral head.

### 2.16 Statistical analysis

All data were analysed by SPSS 21.0 software (IBM, Armonk, NY, USA). Measurement data were presented as the mean ± standard deviation. Differences between two groups were compared by unpaired *t* test, while differences among multiple groups were determined by one‐way analysis of variance (ANOVA). Comparison of the data in each group at different time points was performed using two‐way ANOVA, with *P* < .05 as a level of statistical significance.

## 3 RESULTS

### 3.1 Viability and osteogenic differentiation in of BMSCs were inhibited in ONFH rat models

**Figure 1:**
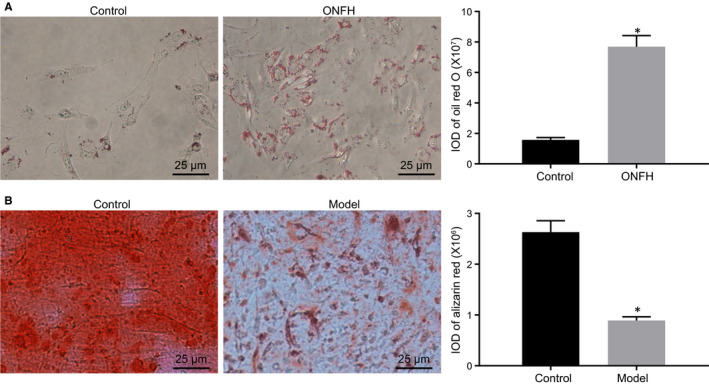
Viability and osteogenic differentiation of BMSCs are reduced in ONFH rats. A, The oil red O staining of BMSCs in normal and ONFH rats (400×; n = 6). B, ARS staining of BMSCs in the normal and ONFH rats (400×; n = 6). *P < .05 vs the normal rats. Measurement data were presented as the mean ± standard deviation derived from at least 3 independent experiments. Differences between two groups were compared by unpaired *t* test, while differences in each group at different time points were compared by two‐way ANOVA

To investigate the role of BMSCs in the ONFH, the viability and differentiation capacity of BMSCs in rats with ONFH were measured. Oil red O staining identified that after 3 weeks of induction, there was stronger deposition in BMSCs of rats with induced ONFH relative to controls (Figure 1A; *P* < .05). In addition, the ARS staining in BMSCs of rats with induced ONFH was weaker than that in the control rats (Figure 1B). Coherently, the cell viability and osteogenic differentiation capacity of BMSCs were decreased in ONFH rats, thus functioning in ONFH.

### 3.2 BMSC‐EVs delivered miR‐148a‐3p to promote osteogenic differentiation

**Figure 2:**
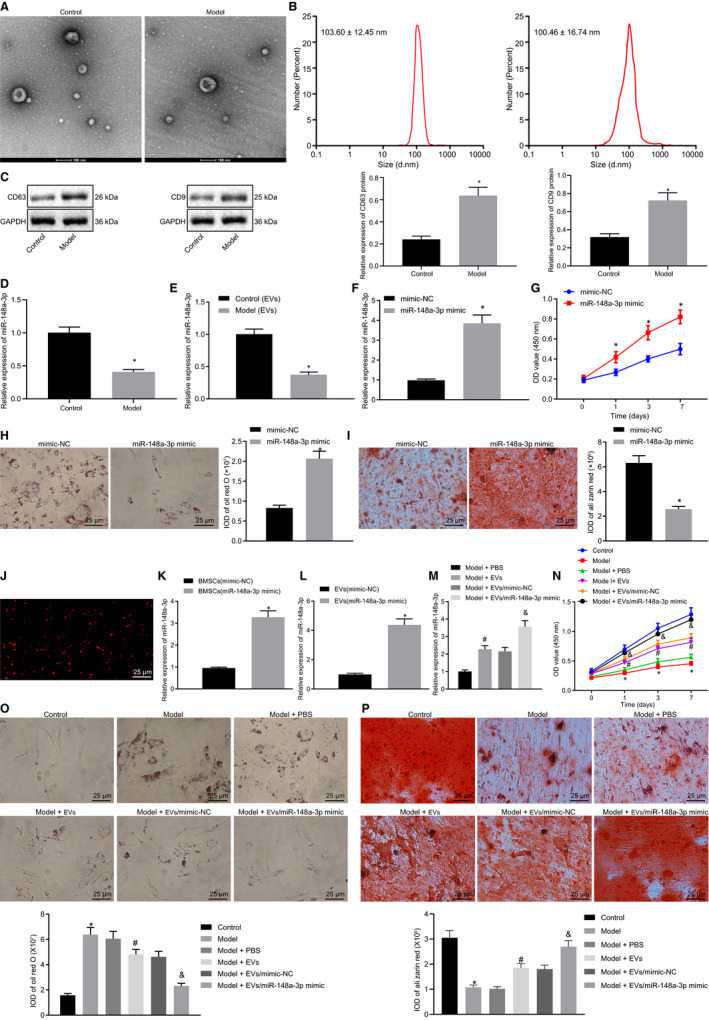
miR‐148a‐3p in BMSC‐EVs increases proliferation and osteogenic differentiation of BMSCs in ONFH rats. A, The particles of EVs detected by TEM. B, The size of particles analysed by DLS (scale bar = 100 nm). C, The expression of CD63 and CD9 in cells and EVs normalized to GAPDH determined by Western blot analysis. D, The expression of miR‐148a‐3p in BMSCs of rats with ONFH detected by RT‐qPCR. E, The content of miR‐148a‐3p in EVs derived from BMSCs of ONFH rats determined by RT‐qPCR. F, Overexpression efficiency of miR‐148a‐3p in BMSCs of ONFH rats. G, The proliferation of BMSCs from ONFH rats after miR‐148a‐3p overexpression analysed by CCK8 assay. H, Oil red O staining of BMSCs from ONFH rats (400×). I, ARS staining of BMSCs from ONFH rats (400×). J, Fluorescent microscopy analysis of DiL‐labelled EVs uptake (400×). K, miR‐148a‐3p expression in BMSCs from normal rats treated with miR‐148a‐3p mimic determined by RT‐qPCR. L, miR‐148a‐3p expression in BMSC‐EV‐miR‐148a‐3p mimic from normal rats determined by RT‐qPCR. M, The expression of miR‐148a‐3p in BMSCs of ONFH rats after treatment with BMSC‐EV‐miR‐148a‐3p mimic determined by RT‐qPCR. N, The proliferation of BMSCs from ONFH rats after treatment with BMSC‐EV‐miR‐148a‐3p mimic analysed by CCK8 assay. O, Oil red O staining of BMSCs from ONFH rats after treatment with BMSC‐EV‐miR‐148a‐3p mimic (400×). P, ARS staining of BMSCs from ONFH rats after treatment with BMSC‐EV‐miR‐148a‐3p mimic (400×). Measurement data were presented as the mean ± standard deviation. *P < .05 vs BMSC‐EVs from the normal rats or people, BMSCs from ONFH rats treated with mimic‐NC or BMSCs from ONFH rats treated with BMSC‐EV‐mimic‐NC; #*P *< .05 vs BMSCs from ONFH rats treated with PBS; &*P *< .05 vs BMSCs from ONFH rats treated with BMSC‐EV‐mimic‐NC. Differences between two groups were compared by unpaired *t* test, while differences among multiple groups were determined by one‐way ANOVA. Comparison of the data in each group at different time points was performed using two‐way ANOVA. n = 6

To further investigate the specific mechanism, EVs derived from BMSCs of normal and ONFH rats were isolated, followed by the evaluation of size, shape and specific proteins of EVs. TEM observation demonstrated that the particles obtained from BMSCs were 100‐200 nm, with uniform membrane structure (Figure 2A). The particles were 103.60 ± 12.45 nm and 100.46 ± 16.74 nm detected by DLS (Figure 2B). Western blot analysis identified that the obtained particles showed significant expression in CD63 and CD9 proteins which were the general markers of EVs (Figure 2C). Taken together, BMSC‐EVs were successfully extracted.

RT‐qPCR detection demonstrated that compared to the normal rats, the expression of miR‐148a‐3p was decreased in BMSCs of ONFH rats (Figure 2D), and that the expression of miR‐148a‐3p in EVs from BMSCs of the ONFH rats was significantly decreased in contrast to EVs from BMSCs of the normal rats (Figure 2E). After miR‐148a‐3p mimic was transfected into BMSCs of ONFH rats, we found that miR‐148a‐3p mimic increased expression of miR‐148a‐3p and promoted the viability of BMSCs in BMSCs of ONFH rats with weakened red O staining and strengthened ARS staining (Figure 2F‐I).

To determine whether BMSC‐EVs of normal rats were internalized into BMSC‐EVs of the ONFH rats, red fluorescent lipophilic dye (DiL) was labelled into EVs secreted by BMSCs of the normal rats in our study. After 2‐hour incubation with BMSC‐EVs of the ONFH rats, Dil‐labelled EVs were transferred to perinuclear region of BMSCs in ONFH rats (Figure 2J).

To explore the effects of EVs‐miR‐148a‐3p on the proliferation and osteogenic differentiation of BMSCs in ONFH rats, BMSC‐EVs of normal rats were treated with miR‐148a‐3p mimic (Figure 2K). EV‐miR‐148a‐3p mimic was isolated (Figure 2L). After that, the isolated EVs were utilized to treat BMSCs of ONFH rats for 24‐48 hours. As shown in Figure 2M, the expression of miR‐148a‐3p was increased in BMSCs of ONFH rats injected with EVs compared to the ONFH rats injected with PBS, whereas that was more significantly elevated in BMSCs of ONFH rats injected with EV‐miR‐148a‐3p mimic compared to the ONFH rats injected with EV‐mimic‐NC. CCK8 detection demonstrated that the proliferation of BMSCs in ONFH rats was decreased compared to the normal rats, while the cell proliferation was increased in BMSCs of ONFH rats injected with EVs or more prominently increased in BMSCs of ONFH rats injected with EV‐miR‐148a‐3p mimic (Figure 2N). Oil red O staining identified that the deposition of oil red O was enhanced in ONFH rats compared to the normal rats, which was blocked by EVs or EV‐miR‐148a‐3p mimic (Figure 2O). Furthermore, ARS staining was decreased in BMSCs of ONFH rats compared to normal rats, which was reversed by treatment with EVs or EV‐miR‐148a‐3p mimic (Figure 2P). In general, miR‐148a‐3p delivered by BMSC‐EVs from normal rats promoted the proliferation and osteogenic differentiation of BMSCs in ONFH rats.

### 3.3 miR‐148a‐3p in BMSC‐EVs promoted the activation of SMAD7‐BCL2 axis by specifically targeting SMURF1

**Figure 3:**
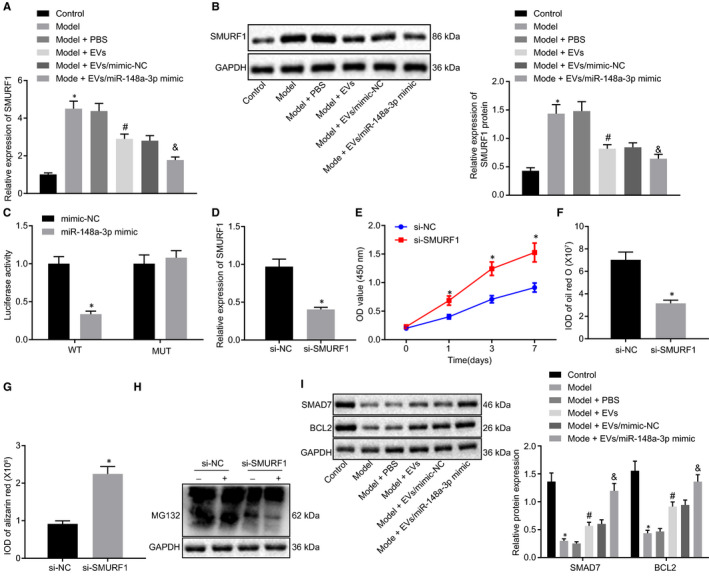
miR‐148a‐3p in BMSC‐EVs targets SMURF1 to elevate the activation of the SMAD7‐BCL2 axis. A, The expression of SMURF1 mRNA in BMSCs from ONFH rats after EV treatment detected by RT‐qPCR. B, The protein expression of SMURF1 in BMSCs from ONFH rats after EV treatment normalized to GAPDH measured by Western blot analysis. C, The binding relationship between miR‐148a‐3p and SMURF1 measured by dual‐luciferase reporter gene assay. D, The silencing efficiency of SMURF1 in BMSCs of the ONFH rats. E, The proliferation of BMSCs in ONFH rats after SMURF1 silencing analysed by CCK8 assay. F, Oil red O staining of BMSCs from ONFH rats after SMURF1 silencing. G, ARS staining of BMSCs from ONFH rats after SMURF1 silencing. H, The protein of ubiquitinated SMAD7 in BMSCs of the ONFH rats depended on MG132 (10 μmol/L) detected by Anti‐HA blot. I, The protein expression of SMAD7 and BCL2 in BMSCs from ONFH rats after EV treatment normalized to GAPDH determined by Western blot analysis. *P < .05 vs BMSCs‐EVs from the normal rats, BMSCs from ONFH rats treated with mimic‐NC, BMSCs from ONFH rats treated with BMSC‐EV‐mimic‐NC, or si‐NC; #*P *< .05 vs BMSCs from ONFH rats treated with PBS; &*P *< .05 vs BMSCs from ONFH rats treated with BMSC‐EV‐mimic‐NC. Differences between two groups were compared by unpaired *t* test, while differences among groups were determined by one‐way ANOVA. Comparison of the data in each group at different time points was performed using two‐way ANOVA. n = 6

There was no direct relation between SMURF1 and ONFH in previous studies, but the models of ONFH rats were induced mainly by steroid hormones.^18^ Androgen as one of the steroid hormones promoted the expression of SMURF1,^19^ which demonstrated that SMURF1 might be related to ONFH. To confirm that miR‐148a‐3p in BMSC‐EVs regulated SMURF1 to prevent ONFH, BMSCs in the ONFH rats were treated with BMSC‐EVs from normal rats. The results demonstrated that the expression of SMURF1 was higher in BMSCs of the ONFH rats than in BMSCs of the normal rats, which was negated by treatment with EVs or EV‐miR‐148a‐3p mimic (Figure 3A,B). The possibly existed binding sites between miR‐148a‐3p and SMURF1 3′UTR were predicted by StarBase. The dual‐luciferase reporter gene assay identified that there was reduced luciferase activity in BMSCs treated with miR‐148a‐3p mimic and psiCHECK2 vector containing WT‐SMURF1 3′UTR (Figure 3C). Above results demonstrated that BMSC‐EV‐miR‐148a‐3p specifically targeted SMURF1.

To further research the effect of SMURF1 on ONFH, SMURF1 was down‐regulated in BMSCs of ONFH rats. As shown in Figure 3D‐G, compared to BMSCs with si‐NC, there were down‐regulated SMURF1, increased proliferation, reduced oil red O staining and elevated ARS staining in BMSCs treated with si‐SMURF1.

It has been reported that SMAD7 interacted with ubiquitin ligases SMURF1 and SMURF2, which further promoted the degradation of SMAD7.^20^ The ubiquitination detection in vivo depended on the proteasome inhibitor MG132 demonstrated that SMURF1 induced the ubiquitination of SMAD7 (Figure 3H). Western blot analysis exhibited that SMAD7 and BCL2 expression were declined in BMSCs of ONFH rats compared to BMSCs of the normal rats, which was neutralized by treatment with EVs or EV‐miR‐148a‐3p mimic (Figure 3I). Above results suggested that BMSC‐EV‐miR‐148a‐3p binding to SMURF1 activated SMAD7 and BCL2 axis in BMSCs from ONFH rats.

### 3.4 miR‐148a‐3p in BMSC‐EVs relieved ONFH

**Figure 4:**
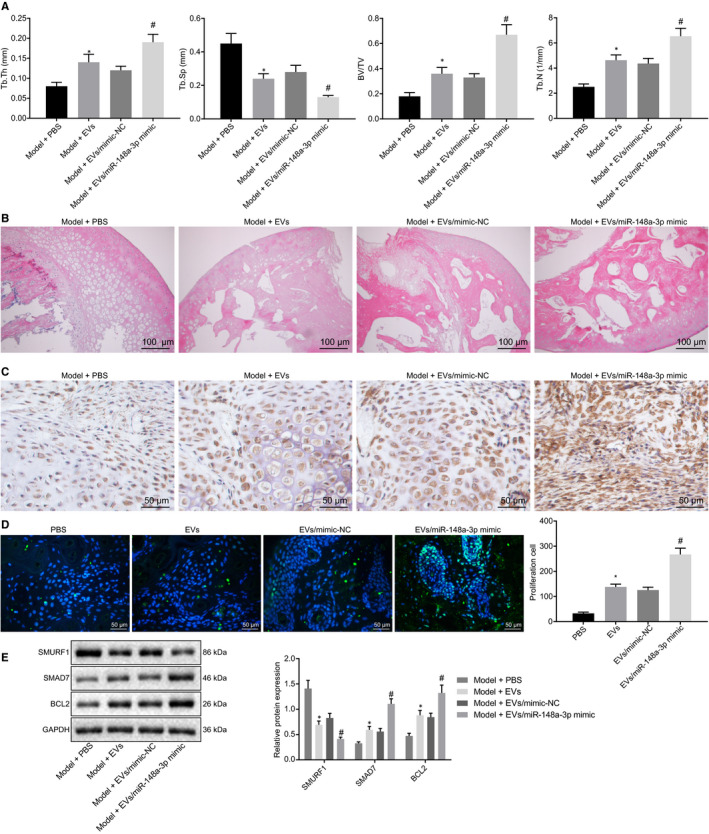
miR‐148a‐3p in BMSC‐EVs attenuates ONFH in rats. A, Tb.Th, Tb.Sp, and BV/TV in ONFH rats after EV treatment measured by quantitative analysis. B, The femoral heads of ONFH rats after EV treatment detected by H&E staining (100×). C, OCN expression in the femoral heads of ONFH rats after EV treatment by immunohistochemical staining (200×). D, The cell proliferation in ONFH rats after EV treatment determined by Ki67 immunostaining (200×). E, The protein expression of SMURF1, SMAD7 and BCL‐2 in femoral heads of ONFH rats after EV treatment normalized to GAPDH measured by Western blot analysis. Measurement data were presented as the mean ± standard deviation. *P < .05 vs the ONFH rats injected with PBS; #*P *< .05 vs the ONFH rats injected with EV‐mimic‐NC. Differences among multiple groups were determined by one‐way ANOVA. n = 6

To investigate the effect of BMSC‐EV‐miR‐148a‐3p on GC‐induced ONFH, the models of ONFH rats were induced using MPS by intramuscular injection. Subsequently, the ONFH rats were injected intravenously with BMSC‐EVs or equal volume of PBS. After 6 weeks, all micro‐CT parameters by quantitative analysis demonstrated that miR‐148a‐3p in BMSC‐EVs played the preventive role in ONFH rats. As demonstrated in Figure 4A, Tb.Th, BV/TV and Tb.N in the ONFH rats injected with EVs (0.14* ± *0.02%, 36.0* ± *5.0%, and 4.63* ± *0.42%) were higher than in the ONFH rats injected with PBS (0.08* ± *0.01%, 18.0* ± *3.0% and 2.51* ± *0.23%), and that were elevated in the ONFH rats injected with EV‐miR‐148a‐3p mimic compared to the ONFH rats injected with EV‐mimic‐NC. In addition, Tb.Sp was lower in the ONFH rats injected with EVs than in ONFH rats injected with PBS (0.24* ± *0.03% vs 0.45* ± *0.06%), while Tb.Sp in the ONFH rats injected with EV‐miR‐148a‐3p mimic was significantly decreased compared to the ONFH rats injected with EV‐mimic‐NC (0.13* ± *0.01% vs 0.28* ± *0.04%).

Consistent with the above results, the detection of haematoxylin‐eosin (H&E) staining identified that the osteonecrosis was apparent, the trabecular bone of the femoral head became thinner or even disappeared, and abnormal morphology exhibited in the ONFH rats injected with PBS. Nevertheless, the bone structure was good, and a small amount of trabecular structure and bone marrow were replaced by necrotic tissues in the ONFH rats injected with EVs. Compared to the ONFH rats injected with EV‐mimic‐NC, the treatment effect was enhanced in the ONFH rats injected with EV‐miR‐148a‐3p mimic (Figure 4B).

Osteocalcin (OCN) was an osteogenesis‐related molecule expressed in osteogenic differentiation and mineralization. OCN immunohistochemical staining demonstrated the osteogenic response of the femoral head was enhanced in the ONFH rats injected with EVs compared to the ONFH rats injected with PBS, and elevated in the ONFH rats injected with EV‐miR‐148a‐3p mimic in contrast to the ONFH rats injected with EV‐mimic‐NC (Figure 4C).

Ki67 immunohistochemistry presented the effects of BMSC‐EVs on the early cell proliferation in femoral head of ONFH rats. The results demonstrated that the cell proliferation was increased in the ONFH rats injected with EVs compared to the ONFH rats injected with PBS, whereas that enhanced cell proliferation was observed in the ONFH rats injected with EV‐miR‐148a‐3p mimic compared with the ONFH rats injected with EV‐mimic‐NC (Figure 4D).

Western blot analysis documented that the protein expression of SMURF1 in femoral head was significantly reduced but the protein expression of SMAD7 and BCL2 was increased in the ONFH rats injected with EVs compared to the ONFH rats injected with PBS. Moreover, decreased SMURF1 protein expression and increased SMAD7 and BCL2 protein expression were showed in the ONFH rats injected with EV‐miR‐148a‐3p mimic compared to the ONFH rats injected with EV‐mimic‐NC (Figure 4E). Taken together, BMSC‐EV‐miR‐148a‐3p improved the symptom of ONFH rats.

## 4 DISCUSSION

ONFH, as one of common and intractable diseases, results in the collapse of the femoral head ultimately, accompanied by the decrease of osteoblast activity in the region of ONFH.^21^ EVs derived from BMSCs were found to regulate steroid‐induced osteonecrosis of the femoral head.^22^ Additionally, miRs are involved in various bone diseases and play a role in the mechanism of ONFH.^23^ Therefore, this study attempted to research a new therapeutic method that miR‐148a‐3p in BMSC‐EVs promoted the proliferation and the differentiation of BMSCs by down‐regulating SMURF1 and up‐regulating SMAD7 and BCL2, thus preventing the development of ONFH.

Initially, we identified that the cell activity and osteogenic differentiation capacity of BMSCs had decreased in ONFH rats. A previous study has reported the decreased osteogenic differentiation and the expand of adipocytes deposition in bone marrow of ONFH.^24^ There are many potential capacities in BMSCs such as the promotion of osteogenesis.^25^ Moreover, BMSCs can be used to treat ONFH in the region of osteonecrosis.^26^ Notably, the primary BMSCs are capable of elevating the cell viability and suppressing the cell apoptosis in the primary osteoblasts.^27^ Additionally, EVs were found to promote the proliferation and differentiation in ONFH. BMSC‐EVs elevate the regeneration of bone and angiogenesis, characterized by the increased trabecular reconstruction and microvascular density.^7^

Subsequently, we identified that the expression of miR‐148a‐3p was poorly expressed in ONFH, and miR‐148a‐3p in EVs derived from BMSCs promoted the proliferation and osteogenic differentiation of BMSCs in ONFH. EVs that are released by cells such as BMSCs and osteoblasts can deliver miRs that are engaged in the development of ONFH.^28^, ^29^ For instance, miR‐122‐5p overexpressed in BMSC‐derived EVs suppresses the expression of RTK signalling antagonist 2 to inhibit the occurrence of ONFH through RTK/Ras/mitogen‐activated protein kinase axis.^30^ Moreover, miR‐26a‐CD34‐EVs strengthened the osteogenic differentiation of BMSCs and the integrity of trabecular bone in glucocorticoids‐induced ONFH.^31^ miR‐148a was found to differentially expressed in BMSC‐EVs.^9^ Notably, miR‐148‐3p is down‐regulated in BMSCs of ONFH mice.^10^ Overexpressed miR‐148a‐3p target gene, lysine‐specific demethylase 6b, could promote the osteoblast differentiation.^32^ The down‐regulation of miR‐148a‐3p diminishes the bovine myoblast proliferation.^33^ Therefore, miR‐148a‐3p in EVs derived from BMSCs improved ONFH.

Furthermore, we detected that SMURF1 was a target gene of miR‐148a‐3p by dual‐luciferase reporter gene assay. The inhibitor of miR‐15b up‐regulates the expression of SMURF1 gene in osteoblast differentiation,^34^ which further supported our results. Moreover, our findings also unravelled that SMURF1 overexpression inactivated SMAD7‐BCL2 axis to inhibit the proliferation and osteogenic differentiation of BMSCs, thus promoting ONFH. A previous study has demonstrated that osteoporosis and osteonecrosis are closely related to ONFH.^35^ Especially, the inhibition of SMURF1 could improve the bone morphogenetic protein (BMP) signalling and the osteogenic differentiation while enhancing the capacity of bone formation in spinal fusion rats, and meanwhile SMURF1 could elevate the capacity of the bone fracture healing.^36^, ^37^ The knockdown of SMURF1 could promote the osteogenesis.^38^ Interestingly, latent membrane protein‐1 and SMURF1 enhance the activity of bone morphogenetic protein by suppressing the ubiquitination of SMADS.^39^ In addition, the knockdown of SMURF1 as an E3 ubiquitin ligaseo of SMAD7 could promote the expression of SMAD7 in mesangial cells.^12^ Another study has reported that up‐regulated SMAD7 has the positive correlation with BCL2 that is the anti‐apoptotic gene in gastric epithelial cells, and the activation of transforming growth factor‐β1 (TGF‐β1), type II TGFβ receptor, p‐smad2/3, SMAD4 and SMAD7 axis activated by SAMC promotes the expression of BCL2, inhibiting the apoptosis of cancer cells.^40^, ^41^ Furthermore, platelet‐rich plasma‐EVs increase the expression of BCL2 through the signalling pathway of Akt/Bad/BCL2, preventing the cell apoptosis in glucocorticoids‐induced ONFH.^17^ SMURF1 down‐regulation promoted BMP2‐induced osteogenic differentiation of BMSCs.^42^ In addition, the inhibition of SMURF1 promotes the proliferation and differentiation of MSCs by regulating JunB.^43^

In conclusion, the key findings obtained from the current study demonstrated the regulatory role of miR‐148a‐3p in BMSC‐EVs interacting with SMURF1, SMAD7 and BCL2 in ONFH. miR‐148a‐3p in BMSC‐EVs increased the proliferation and differentiation of BMSCs by suppressing SMURF1 and promoting the SMAD7‐BCL2 axis, thereby preventing ONFH. These findings suggested that miR‐148a‐3p in BMSC‐EVs may serve as a promising therapeutic target in ONFH. At present, the effects and mechanisms of miR‐148a‐3p/SMURF1/SMAD7/BCL2 axis remain scantly identified in the prevention of ONFH, and we will further discuss the underlying rules that govern their interaction in our further work, which might increase the feasibility and safety of its therapy in clinical applications.

## CONFLICT OF INTEREST

The authors declare that there is no conflict of interest.

## Author Contribution

**Shengxiang Huang:** Conceptualization (equal); Formal analysis (equal); Supervision (equal); Writing‐original draft (equal). **Yaochun Li:** Data curation (equal); Investigation (equal); Resources (equal). **Panfeng Wu:** Methodology (equal); Software (equal); Validation (equal). **Yongbing Xiao:** Investigation (equal); Visualization (equal); Writing‐review & editing (equal). **Ningbo Duan:** Formal analysis (equal); Resources (equal); Writing‐original draft (equal). **Jing Quan:** Conceptualization (equal); Resources (equal); Validation (equal). **Wei Du:** Data curation (equal); Formal analysis (equal); Writing‐original draft (equal). 

## Data Availability

The data that support the findings of this study are available from the corresponding author upon reasonable request.
